# Nano-Spray-Drying of Cyclodextrin/Ibuprofen Complexes with Aerosolization-Enhancing Additives for Pulmonary Drug Delivery

**DOI:** 10.3390/ijms26094320

**Published:** 2025-05-01

**Authors:** Anett Motzwickler-Németh, Endre Körmendi, Árpád Farkas, Ildikó Csóka, Rita Ambrus

**Affiliations:** 1 Institute of Pharmaceutical Technology and Regulatory Affairs, Faculty of Pharmacy, University of Szeged, 6720 Szeged, Hungary; nemeth.anett@szte.hu (A.M.-N.); endre2000@gmail.com (E.K.); csoka.ildiko@szte.hu (I.C.); 2 HUN-REN Centre for Energy Research, Environmental Physics Department, Institute for Energy Security and Environmental Safety, Konkoly-Thege Miklós Street 29-33, 1121 Budapest, Hungary; farkas.arpad@ek.hun-ren.hu

**Keywords:** ibuprofen, sulfobutylether-β-cyclodextrin, (2-Hydroxy-3-N,N,N-trimethylamino)propyl-beta-cyclodextrin chloride, targeted delivery, alternative administration route, dry powder inhaler, inclusion technology, drug delivery

## Abstract

Cyclodextrins (CDs) enhance the solubility of poorly water-soluble drugs like ibuprofen (IBU), making them promising carriers for pulmonary drug delivery. This route lowers the required dose, minimizing side effects, which could be beneficial in treating cystic fibrosis. In this study, a nano-spray-drying technique was applied to prepare CD/IBU complexes using sulfobutylether-β-cyclodextrin (SBECD) or (2-Hydroxy-3-N,N,N-trimethylamino)propyl-beta-cyclodextrin chloride (QABCD) as carriers as well as mannitol (MAN) and leucine (LEU) as aerosolization excipients. Various investigation techniques were utilized to examine and characterize the samples, including a Master Sizer particle size analyzer, scanning electron microscopy (SEM), X-ray powder diffraction (XRPD), differential scanning calorimetry (DSC), and Fourier-transform infrared spectroscopy (FT-IR). We applied in vitro Andersen Cascade Impactor measurements and in silico simulation analysis to determine the sample’s aerodynamic properties. We also performed in vitro dissolution and diffusion tests. Applying formulations with optimal aerodynamic properties, we achieved an improved ~50% fine particle fraction values based on the Andersen Cascade Impactor measurements. The in vitro dissolution and diffusion studies revealed rapid IBU release from the formulations; however, the QABCD-based sample exhibited reduced membrane diffusion compared to SBECD due to the formation of electrostatic interactions.

## 1. Introduction

**Figure 1:**
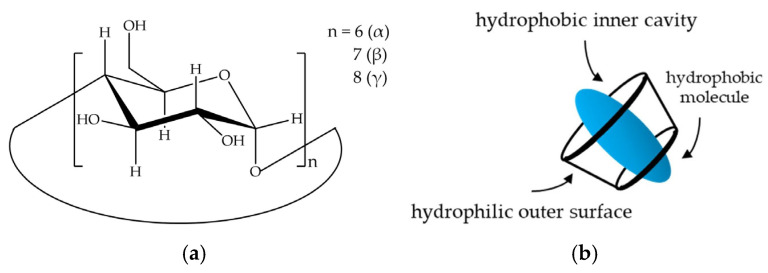
The chemical formula of CDs (**a**), along with a schematic representation of the molecule (**b**).

The donut-shaped cyclodextrins (CDs) are cyclic oligosaccharide molecules that are usually formed by six (αCD), seven (βCD), or eight (γCD) (Figure 1a) D-glucopyranose units. The inner cavity of the CD is formed by the hydrophobic carbon skeleton of the glucopyranonose monomers; therefore, the interior of the CD is apolar [1] (Figure 1b). Because of this unique structural property, CDs are mainly used as solubilizers of molecules that are not water-soluble [2,3,4,5]. Furthermore, CDs are able to influence other physicochemical properties of molecules and are therefore widely applied as stabilizing agents [2,3,4,5,6], permeability enhancers [6,7,8], or even for masking undesirable taste characteristics [9]. Since CDs are edible and non-toxic for human consumption [10], they have become widely used in the food and pharmaceutical industries.

βCD is the most extensively researched and utilized CD type [11], primarily due to its cost-effectiveness since this type has the lowest production price. However, the primary limitation is its relatively low solubility in water [12]. The limited solubility of βCD in aqueous environments poses a significant challenge for its industrial and pharmaceutical applications. To address this challenge, the -OH groups on the βCD can be chemically modified, resulting derivatives of improved properties [13]. CD derivatives are utilized as excipients in commercially available pharmaceutical formulations. Hydroxypropyl-β-cyclodextrin (HPBCD) and sulfobutylether-β-cyclodextrin (SBECD) are the most widely applied CD-based drug delivery agents among the βCD derivates. The majority of currently available formulations are administered orally, including tablets, capsules, syrups, chewing gum, and mouthwashes, or via the intravenous route. However, products intended for other routes of administration, such as ocular eye drops, vaginal gels, suppositories, and transdermal ointments and creams, are also commercially available [14,15]. By enhancing the aqueous solubility of certain compounds, CDs improve the bioavailability of active pharmaceutical ingredients [16]. This increase in bioavailability can allow for a reduction in the required dosage, potentially minimizing or preventing the side effects associated with higher concentrations of drugs.

In addition to the use of CDs, alternative administration routes such as pulmonary [17,18], ocular [19], and nasal [20] delivery can also be employed to reduce the required drug dosage. These routes offer potential for more efficient drug absorption by avoiding the first-pass mechanism, which can further minimize the amount of active ingredients needed, potentially reducing side effects. Because of the unique physiological features of the lungs [21] (large alveolar surface area, extensive vascularization, and thin alveolar epithelium), pulmonary administration of drugs with local treatment can significantly enhance drug absorption. Since extravascular lung water, which helps maintain tissue moisture, is limited in normal lungs [22], achieving optimal absorption of active substances requires increasing the solubility of poorly soluble compounds. The most important requirements for particles intended for inhalation concern the particle size, which are as follows: Particles that reach the deep lung areas should have a diameter of 1 to 5 µm [23]. The upper respiratory tract receives particles larger than 10 µm [24], whereas the exhalation process takes place for particles smaller than 1 µm. Researchers frequently use key metrics like fine particle fraction (FPF) and median mass aerodynamic diameter (MMAD) to describe aerodynamic characteristics of the samples. These metrics serve as essential indicators for investigating and analyzing the aerosolization performance.

As several clinical investigations highlight the advantages of long-term high-dose ibuprofen (IBU) administration in patients who suffer from the genetic disorder cystic fibrosis [25,26,27], the use of IBU through the pulmonary route as a dry powder inhalation (DPI) product is becoming a more and more attractive topic among researchers. The pulmonary dose required for IBU tablets used in cystic fibrosis treatment can be significantly reduced [28] due to IBU’s low water solubility and bypassing of the first-pass metabolism, enabling rapid and effective therapy while minimizing side effects. It is worth noting that caution is advised when administering IBU to children with respiratory diseases, as research indicates a potential association between its use and the exacerbation of asthma symptoms [29]. According to these results, IBU may trigger a worsening of symptoms in patients with pre-existing asthma, highlighting the persistent risk of respiratory adverse effects in children. While the solubility of IBU in water is only 0.021 mg/mL [30], numerous studies have aimed to enhance its solubility in aqueous environments. One particularly effective approach involves complexation with CDs, which not only improves solubility but also enhances the dissolution rate, stability, and bioavailability of the drug. In the scientific literature, IBU is most commonly complexed with β-type CDs [31,32,33]. In these complexes, the less polar part of the IBU molecule is typically embedded within the hydrophobic cavity of the host, while the drug retains the ability to freely enter and exit the CD structure. According to most studies, IBU forms a 1:1 molar ratio IBU:CD complex with βCD [34,35,36] and its derivates. Nevertheless, different stoichiometries, such as 1:2 [37], 1:3 [38], or 2:3 [39], have also been reported in the literature, indicating that the complexation behavior of IBU even with unsubstituted βCDs varies depending on the experimental conditions. It is important to note that the formation of these inclusion complexes is influenced by several factors, including pH [40] and temperature [38].

A variety of techniques, including co-precipitation, freeze-drying, kneading, melting, neutralization, grinding, and spray-drying are applicable to produce CD–guest complexes [41]. Among these methods, spray-drying is one of the most widely utilized techniques, while it is one of the most prevalent bottom-up approaches in the pharmaceutical industry. It enables the simple and fast preparation of complexes of precise size and morphology, making the final products suitable for many applications. Despite its numerous advantages, one major challenge of spray-drying is the formation of amorphous products with low thermodynamic stability, which are prone to crystallization, making the long-term stability of the resulting powders difficult to ensure. While spray-drying is a common formulation technique, some applications require more accurate techniques. A possible solution is offered by the gentler nano-spray-drying technique, a formulation process that is becoming more and more popular in the literature. The unique feature of this technology lies in its vibrating mesh spray system, which generates fine droplets, enabling the production of submicron-sized powder particles. The nano-spray-dryer produces particles with a more uniform size distribution and smaller size range than conventional spray-dryers [42], offering advantages for pulmonary drug delivery applications [17,43,44].

We recently reported on nano-spray-dried differently charged β-cyclodextrin-based meloxicam potassium-containing nasal powders [8], where the obtained particles showed a narrow size distribution, spherical shape, and their particle size was well suited to the requirements of pulmonary delivery. In our previous study, we investigated the complexation of the active pharmaceutical ingredient meloxicam-potassium with SBECD, (2-Hydroxy-3-N,N,N-trimethylammonio)propyl-β-cyclodextrin chloride (QABCD), and βCD for nasal powder formulation development. Our findings suggested that the charge of the CDs, whether negative or positive, may influence the drug’s permeability. However, the underlying mechanisms and the direction of these effects remained unclear. Accordingly, the present study focuses on two types of CDs with opposite charges to better elucidate their individual effects. Based on these insights, we propose that further investigation into nano-spray-dried SBECD and QABCD formulations for pulmonary delivery could be beneficial. Although the number of publications is steadily increasing both in the field of CD complexation and in DPI formulation development, where nano-spray-drying is applied as a formulation strategy, we found only one available publication in which CD complexes were prepared with the aforementioned technology for pulmonary delivery purposes [45]. Adel et al. applied the nano-spray-drying technique to develop curcumin-containing proliposomes, employing HPβCD as carrier. Proliposomes are dry powders consisting of lipid vesicles that encapsulate the drug within their bilayer structure. Upon hydration under controlled conditions, proliposomes rapidly transform into liposomes. The optimized formulation, incorporating lecithin and cholesterol in a 1:1 *w*/*w*% ratio, demonstrated enhanced aerodynamic properties, as evidenced by an FPF of 54.3 ± 8.2% and an MMAD of 2.10 ± 0.26 µm.

In summary, enhancing the solubility of poorly water-soluble active compounds through complexation with CDs may be advantageous, particularly when using pulmonary administration as the delivery route. The challenge is that CDs alone do not exhibit optimal aerosolization properties, resulting a low FPF% of the formulations that reach the lungs [46] and achieve therapeutic effect. Therefore, it is advisable to improve their aerodynamic properties, which can be accomplished by controlling particle size and with the use of additional excipients [47,48,49,50] that enhance aerosolization properties. Among these materials, many amino acids [51], magnesium stearate, lactose [52], trehalose, and mannitol (MAN) [53] have already been reported in the literature. According to the literature, aerosolization enhancer leucine (LEU) is the most often combined with CDs [47,49,50]. Despite numerous reports of the advantageous combination of multiple excipients [52,53,54], we have not found any data examining the combination of several excipients in addition to SBECD or QABCD. Notably, C. Molina et al. reported that a MAN/LEU combination significantly improved aerosolization performance, increasing the FPF% by 52.96 ± 5.21% [55] and offering a potential lactose-free alternative for patients with respiratory conditions and lactose intolerance. Malamatari et al. optimized a pulmonary formulation using a 1:1:0.5 IBU:MAN:LEU weight ratio, achieving an FPF of 68.55 ± 3.8% [54]; this ratio was later employed by P. Party et al. in combination with the stabilizing polymer polyvinyl alcohol and resulted in an FPF% of 70.65 ± 2.47% [17]. Furthermore, N. Mohtar et al. demonstrated that increasing the LEU content improved the aerosolization of SBECD-based fisetin formulations, with 20 *w*/*w*% LEU increasing the FPF% 2.3-fold [56]. However, due to the typically low proportion of low-molecular-weight NSAIDs relative to high-molecular-weight CDs, minimizing excipient content is essential to maximize active pharmaceutical ingredient loading. Based on these findings, the combined use of MAN and LEU with CD carriers represents a promising strategy for the development of dry powder inhalation formulations while ensuring adequate drug loading.

Although CDs hold significant promise for pulmonary drug delivery, limited data are available on their cytotoxicity in lung cells. Studies indicate that hydroxypropyl-β-cyclodextrin (HPβCD), hydroxypropyl-α-cyclodextrin (HPαCD), and γCD exhibit low toxicity in Calu-3 airway epithelial cells, whereas randomly methylated β-cyclodextrin (RMβCD) is more cytotoxic, inducing membrane damage and cell death at lower concentrations [57]. Among natural CDs, γCD appears least toxic; however, toxicity is not only determined by ring size but also by chemical substitution. For instance, HPβCD is safer than unsubstituted βCD, while RMβCD is more harmful. Therefore, cytotoxicity must be evaluated for each CD type, considering substitution type, substitution degree and position, CD concentration, the encapsulated drug, complex stability, and additional components [58,59,60]. Substituted CDs, in particular, require more detailed investigation. Data on the cytotoxicity of SBECD in lung cell lines are limited, while the cytotoxic profile of QABCD remains unexplored. It has been reported that complexation of fisetin with SBECD did not modify its cytotoxicity in A549 cells [56]; however, its safety profile for pulmonary administration remains insufficiently explored, highlighting the need for comprehensive investigation and regulatory evaluation of CD-related cytotoxicity in inhalation therapies.

The aim of the present study was to produce SBECD- and QABCD-containing carriers via nano-spray-drying for pulmonary delivery, using MAN and LEU as aerosolization enhancers. Our objective was to identify the optimal MAN/LEU ratio for pulmonary administration while minimizing the use of aerosolization excipients and maximizing the drug content in the formulation with CDs as this area had limited existing research. As the model active ingredient, IBU was chosen with poor water solubility, which may provide an excellent opportunity for pulmonary treatment of cystic fibrosis with optimized formulations with CDs.

## 2. Results and Discussion

### 2.1. Determination of CD Concentration and CD/IBU Molar Ratio

**Figure 2:**
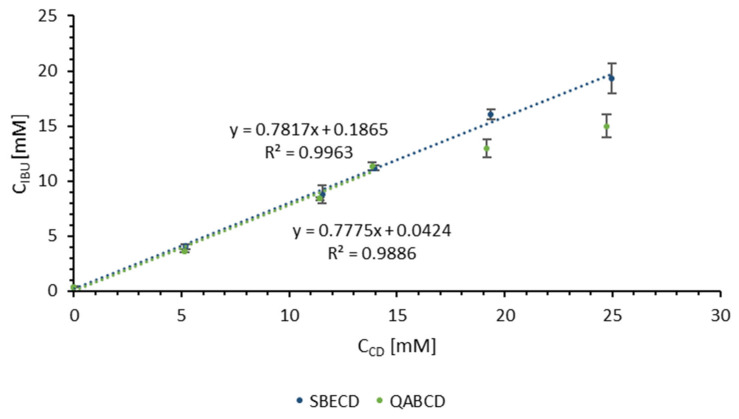
Phase solubility profile of IBU when complexed with SBECD or QABCD in water at 25 °C.

The IBU concentrations measured in CD solutions of different concentrations at 25 °C were illustrated in Figure 2. Comparing the phase solubility test obtained for SBECD and QABCD, there was a difference in the two phase solubility profiles. Although in both cases the phase solubility curve can be classified as main type A as the apparent solubility of the substrate increases with increasing CD concentrations, SBECD+IBU appeared to be of type A_L_ while the QABCD+IBU of type A_N_ [61]. On the one hand, this classification is supported by the solubility profile of SBECD, which demonstrated a linear increase in IBU solubility as a function of solubilizer concentration. Applying linear fitting over the entire dataset, the regression coefficient (R^2^) was found to be 0.9963, indicating excellent linearity within the investigated concentration range. In contrast, in the case of QABCD, the data indicate a reduced efficiency in IBU solubilization at higher QABCD concentrations, as evidenced the presence of a breakpoint in the profile at approximately 15 mM concentration. However, in both cases involving CD, a linear fit could be applied to the curves up to a concentration of 15 mM. Consequently, the CD concentration was set at 15 mM, as this represents the maximum concentration at which a comparable ratio of complex formation between CDs and IBU could still be achieved. After fitting and determining the equations of the lines, using Equations (1)–(3), the K (stability constant), the CE (complexation efficiency) and the drug/CD molar ratio of the complexes were calculated, respectively. The intrinsic solubility (S_0_) of IBU was found to be 0.37 ± 0.05 mM. The results obtained are presented in Table 1.

The stability constant in both cases was close to 10,000 M^−1^, which falls within the range reported in the literature for the stability constants of CD/IBU complexes when IBU was in unionized form [40]. The stability constant showed that in the selected ranges, the affinity of the drug was identical for both CDs. The observed similarity indicates comparable drug affinity between the two CDs. This implies that factors such as differences in charge did not influence the efficiency of complex formation, as evidenced by the closely aligned CE values in Table 1. The comparable affinity can likely be attributed to the presumed uncharged, apolar form of IBU under the experimental conditions. At higher pH levels, where IBU exists in its deprotonated form, a stronger affinity would be anticipated with QABCD due to favorable electrostatic attraction between oppositely charged species. Conversely, a reduced affinity would be expected with SBECD, as a result of electrostatic repulsion between similarly negatively charged materials. The CE values were used to calculate the SBECD or QABCD/IBU ratio. Based on the results, the CD/IBU molar ratio was determined to be 1.3:1 at a CD concentration of 15 mM. This molar ratio indicates the average number of IBU molecules associated with 1 mole of CDs under our experimental conditions, and it probably results from the coexistence of multiple equilibria involving complexes of varying stoichiometries (e.g., 1:1 and 1:2). Due to the complexity of these equilibria, precise identification of the stoichiometric distribution requires advanced structural analysis techniques and molecular docking [62,63]. Based on our current knowledge, we hypothesize that the dominant CD/IBU stoichiometry in our systems was 1:1, coexisting with complexes of other stoichiometries. However, further studies would be necessary for a more detailed interpretation. The experimentally determined CD/IBU ratios were used during sample preparation.

### 2.2. Determination of Particle Size

With CD concentrations determined based on a phase solubility study, samples with the composition shown in Table 5 were nano-spray-dried, then the average particle size of the prepared powders was examined by laser diffraction. The results are presented in Table 2. Since the previous results showed that 50% of the volume distribution of all prepared formulations was between 1 and 5 µm, which is the size range suitable for pulmonary delivery [23], all formulations were considered suitable for application through the lungs.

### 2.3. Investigation of Aerodynamic Properties of Formulations

**Figure 3:**
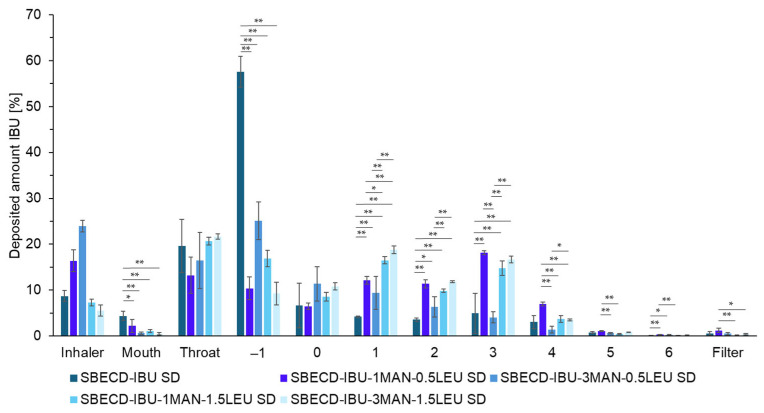
In vitro aerodynamic distribution of the nano-spray-dried samples with SBECD. Data are means ± SD (*n* = 3 independent measurements). Level of significance: * *p* < 0.05, ** *p* < 0.01.

In order to select the optimal formulation, we examined the aerodynamic properties of the formulations in vitro using an Andersen Cascade Impactor. The results of the experiment are presented in Figure 3 and Table 3. It was observed that the formulation without MAN and LEU exhibited a very low FPF value of 15%, which indicated poor aerodynamic performance of the DPI. In contrast, all formulations containing aerosolization excipients demonstrated superior performance, and the SBECD-IBU-1MAN-0.5LEU SD formulation showed particularly notable results. It is worth noting that others have reported the same ratio of components [17,54] in the absence of higher amounts of CDs. Interestingly, the same ratio proved effective even in the presence of SBECD, despite the aerosolization excipients being incorporated in such a low mass fraction relative to the overall formulation. In summary, we concluded that the LEU- and MAN-to-drug ratio, in combination with CDs, are the most significant factors influencing the FPF%. Furthermore, increasing the quantity of excipients adversely affected the aerosolization performance of the formulations.

**Figure 4:**
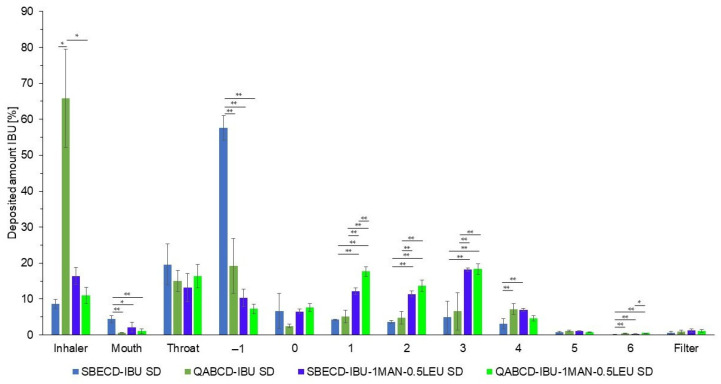
In vitro aerodynamic distribution of the nano-spray-dried samples without aerosolization enhancers compared to optimized formulations containing SBECD and QABCD. Data are means ± SD (*n* = 3 independent measurements). Level of significance: * *p* < 0.05, ** *p* < 0.01.

The formulation with a 50.05 ± 1.05 FPF% value was also prepared using another type of CD, QABCD, to determine whether the ratio of the aerosolization excipient is universally applicable across different CDs or if it was specifically effective with SBECD. The results are presented in Figure 4 and Table 4, which compare formulations with and without the aerosolization excipient, as well as the two types of CDs. No significant difference was found between the two types of CDs in terms of either FPF or MMAD values, meaning that the MAN/LEU ratio used, when combined with both types of CDs, greatly increases the aerosolization performance of the formulations. It is worth noting that QABCD without the presence of MAN or LEU excipients exhibited a higher FPF% compared to SBECD (Table 4). The reason for this difference, however, remains unclear and would require further experimental investigation, as multiple factors may contribute to the observed difference in FPF% values. Clear distinctions exist between the two types of CDs, including their different charges, the density of functional groups (the degree of substitution was approximately 6.0 for SBECD and 3.3 for QABCD), and also the relative length of these substituents on the CD rings. Such structural features may influence the formation of solid dispersions and the morphology of the resulting particles, both of which directly affect aerosolization behavior, similarly to the influence of the CDs’ charge. In order to evaluate the specific role of substituent length, charge, and density on aerosol performance, further studies would be necessary.

Although an FPF of approximately 50% is often associated with efficient lung deposition, there is no universally accepted standard or cut-off value to define an optimal FPF. Among recent studies on pulmonary delivery of IBU, the most favorable FPF results were reported by Party et al., who achieved FPF values of 70.65 ± 2.5% [17] and, in another study, 63.62 ± 3.0% [64], using MAN, LEU, and polyvinyl alcohol or poloxamer 188 as stabilizing agents, respectively. In contrast, other formulations developed for IBU delivery via inhalation over the past five years yielded lower FPF values than our current formulations (SBECD-IBU-1MAN-0.5LEU SD and QABCD-IBU-1MAN-0.5LEU SD), specifically 47.2 ± 4.2% [18], 37.1 ± 3.8% [65], and 45.6 ± 1.6% [66], indicating that our results are notable.

**Figure 5:**
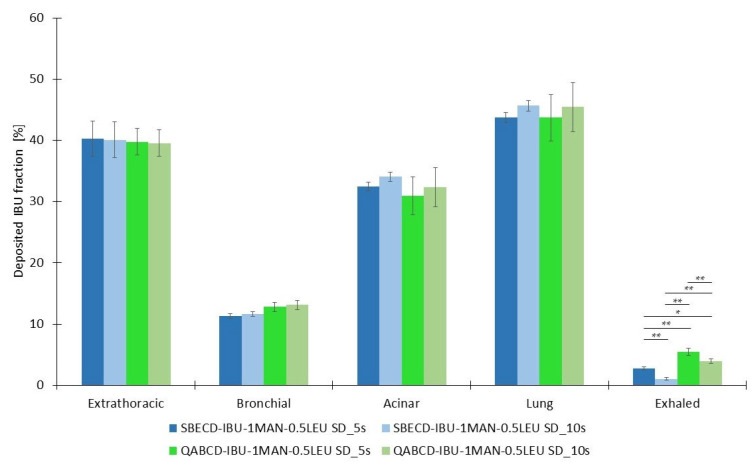
In silico aerodynamic results of the dry powders of SBECD-IBU-1MAN-0.5LEU SD_5s, SBECD-IBU-1MAN-0.5LEU SD_10s, QABCD-IBU-1MAN-0.5LEU SD_5s, and QABCD-IBU-1MAN-0.5LEU SD_10s. Data are means ± SD (*n* = 3 independent measurements). Level of significance: * *p* < 0.05, ** *p* < 0.01.

Combining in vitro aerodynamic investigations with in silico simulation techniques provides a more comprehensive and perceptive study. Different breath-holding times were established during the in silico analysis of the deposited fractions of the formulations, namely 5 s and 10 s. In addition to comparing inhalation times, the distribution of the deposited samples in the bronchial and acinar pulmonary regions, as well as in the extrathoracic airways, are presented in Figure 5. The 10 s breath-hold appeared to decrease both the exhaled fraction and the deposition in the upper airways for both formulations, potentially enhancing particle deposition in the bronchial and acinar regions. However, these differences did not reach statistical significance, indicating that breath-hold duration did not have a measurable impact on deposition patterns under the tested conditions. Additionally, no significant differences were observed in the pulmonary deposition efficiency between the two types of CDs, suggesting that both could be suitable for pulmonary delivery based on their aerodynamic properties. Although statistically significant differences (*p* < 0.05 or *p* < 0.01) were observed among the tested samples in terms of the exhaled fraction of the emitted dose, the absolute values of the exhaled fractions remained low. Therefore, the observed statistical significance is unlikely to have practical or physiological relevance. This indicates the potential suitability of the investigated complexes for use in dry powder inhaler systems. Our findings also indicate that a shorter inhalation time of 5 s is sufficient to achieve effective lung deposition, which could be particularly advantageous for patients with compromised respiratory function.

### 2.4. Characterization of the Optimized Formulations

**Figure 6:**
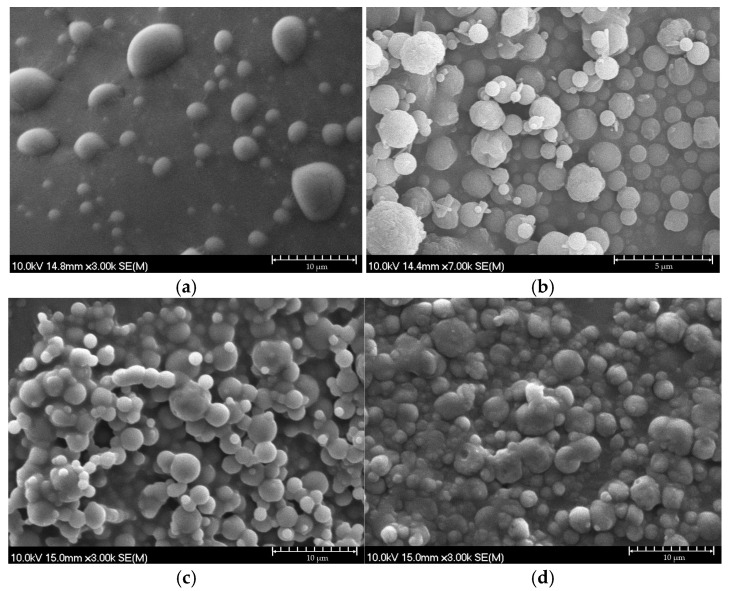
SEM micrographs of sample SBECD-IBU SD (**a**), SBECD-IBU-1MAN-0.5LEU SD (**b**), QABCD-IBU SD (**c**), and QABCD-IBU-1MAN-0.5LEU SD (**d**).

Based on the SEM images (Figure 6a–d), the nano-spray-dried samples without aerosolization excipients, containing only CD and IBU, showed a smooth surface (Figure 6a,c), as our research group has reported in several previous works [8,67]. This spherical morphology can be achieved from aqueous solutions through spray-drying when the drying process is properly set. However, the samples containing MAN and LEU showed a slightly wrinkled surface (Figure 6b–d), a characteristic that enhances their suitability for pulmonary delivery [68,69]. The alteration in surface morphology is attributed to the presence of LEU, as surface wrinkling has been observed in various formulations containing the aforementioned excipient. Notably, this study highlights the interesting finding that even a small amount of LEU compared to the total weight of the formulation can influence the surface structure of the particles.

**Figure 7:**
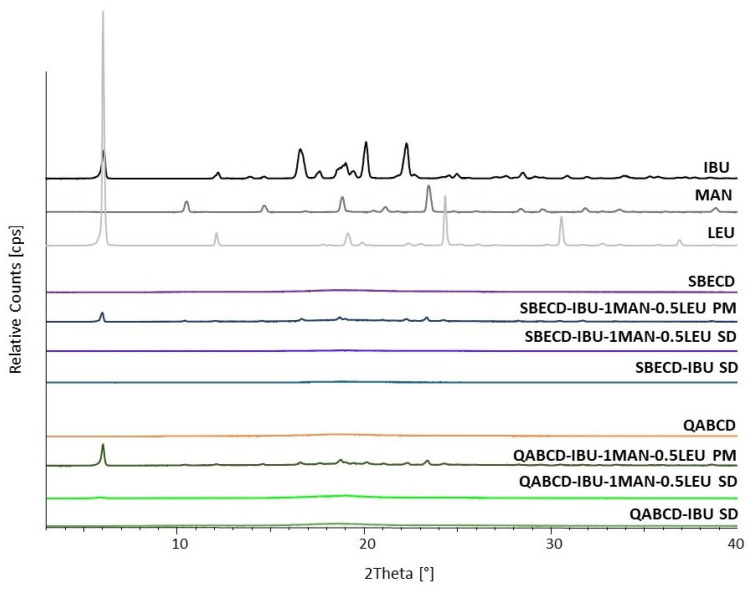
XRPD diffractograms of raw materials, spray-dried powders, as well as the physical mixtures (PMs) corresponding to the selected formulations.

XRPD was utilized to examine the crystalline state of the formulations. The XRPD patterns of the raw materials, physical mixtures (PMs), and the spray-dried (SD) formulations are shown in Figure 7. The diffractograms of the raw materials indicated that IBU, MAN, and LEU possessed crystalline structure, while both of the modified CDs exhibited an amorphous state. The characteristic reflections of IBU [70] appeared at 6.11°, 12.21°, 13.9°, 14.69°, 16.67°, 17.63°, 19.04°, 19.43°, 20.17°, 22.32°, 22.70°, 24.60°, 24.97°, 25.65°, 27.63°, and 28.53° 2Ѳ values. Based on data from the Cambridge Structural Database, the diffraction pattern of the IBU corresponds to Polymorphic Form I, which is the most thermodynamically stable [71] and thus the most commonly observed crystalline form, as evidenced by the presence of its characteristic reflections. Compared with the diffractograms of different polymorphic modifications of mannitol [54,72], the MAN we used was a β polymorphic form. The corresponding diffraction peaks appeared at 10.57°, 17.65°, 16.9°, 21.19°, 23.5°, and 29.61° 2Ѳ values. LEU showed its characteristic reflections [54] at 6.05°, 12.15°, 24.41°, 30.62°, and 36.84° 2Ѳ values. For the PMs, the peaks underwent significant reductions due to the amorphous nature of the CDs, although the main characteristic reflections of crystalline materials were still observable at low intensities. The patterns of the PMs were completely identical for both CDs. The characteristic reflections were visible at 2Ѳ values of 6.05°, 10.46°, 12.09°, 14.58°, 16.73°, 18.76°, 18.99°, 20.70°, 21.02°, 22.26°, 22.71°, 23.39°, 24.30°, 28.25°, 29.44°, 30.51°, 31.70°, 33.39°, 34.07°, 35.59°, 36.72°, and 38.59°, which correspond to the previously mentioned main reflections. In the case of the SD products, the intensities of these characteristic peaks disappeared, indicating that nano-spray-drying reduced the original crystallinity of IBU and the aerosolization excipients. This reduction in crystallinity is particularly beneficial for pulmonary administrations, as the resulting increased dissolution rate significantly enhances bioavailability, thereby improving the therapeutic efficacy of the formulation.

**Figure 8:**
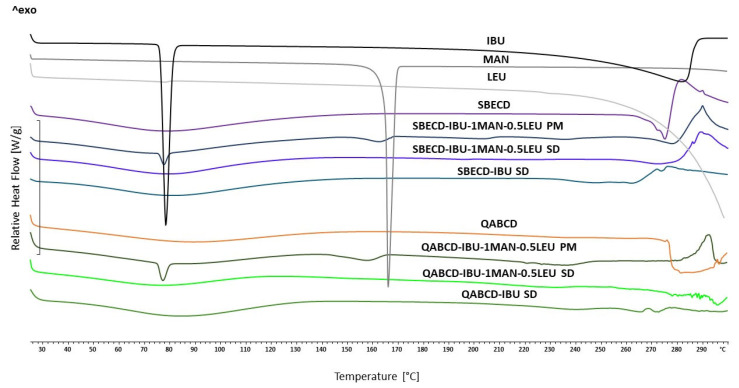
DSC thermal profiles of raw materials, spray-dried powders as well as the physical mixtures corresponding to the selected formulations.

DSC was employed to investigate the thermal behavior of the formulations. The thermograms of the raw materials, PMs, and the SD formulations are shown in Figure 8. The DSC curve of IBU showed two endothermic peaks at 78.4 °C and 282.5 °C, which were associated with the IBU’s melting point and degradation. The melting point of crystalline MAN appeared at 166.0 °C, while the melting point of LEU did not appear in the temperature range we examined. The CDs exhibited endothermic bands below 150 °C, corresponding to the loss of adsorbed and absorbed water. The endothermic peaks also appeared in the case of PMs, together with the characteristic endothermic peaks of the raw materials, although slightly shifted to lower temperatures. The melting point of IBU appeared at 77.7 °C (SBECD-IBU-1MAN-0.5LEU PM) and 77.1 °C (SBECD-IBU-1MAN-0.5LEU PM), while the melting point of MAN appeared at 162.6 and 157.6 °C, respectively. An exothermic peak was observed in both cases between 280 °C and 295 °C, which is likely a consequence of heat release caused by the decomposition of the samples. The thermograms of the SD samples showed distinct differences compared to PMs. Notably, the melting points of raw IBU and MAN were absent, suggesting the amorphization of the materials. This finding aligns with the XRPD results, which further support the absence of crystallinity in the formulations. Following the XRPD analysis, this provided additional support for our hypothesis that the formulations could achieve a rapid dissolution profile and enhanced bioavailability. Furthermore, no exothermic peak between 280 °C and 295 °C was observed in any of the SD samples, suggesting thermal stability of the complexes.

**Figure 9:**
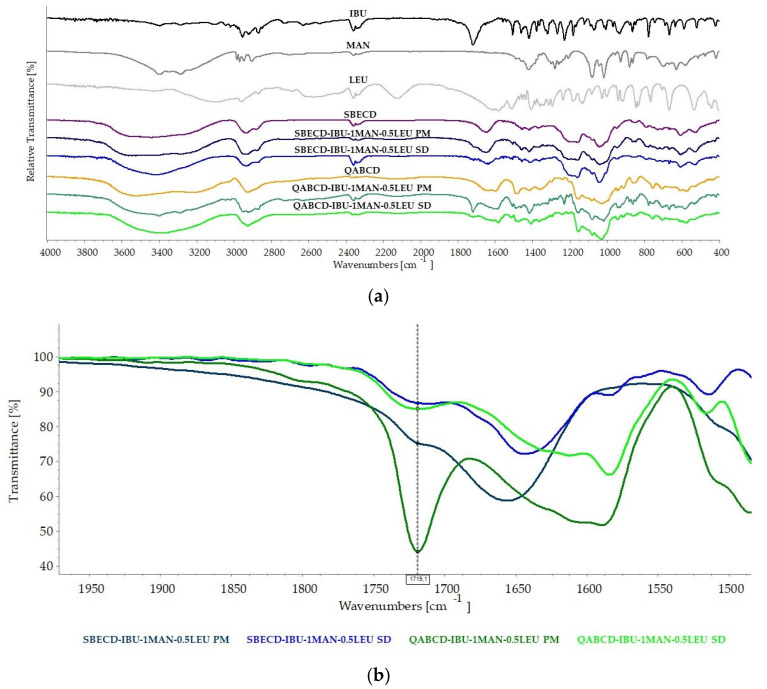
FT-IR spectra of raw materials, spray-dried powders, as well as the physical mixtures corresponding to the selected formulations (**a**). FT-IR spectral analysis of -COOH functional groups in spray-dried powders and physical mixtures associated with the selected formulations (**b**).

FT-IR was used to examine the intermolecular interactions between the compounds. Figure 9a presented the measured spectra. The spectra of PMs closely align with CDs, primarily due to their high mass. Peaks associated with other components were visible in certain regions. Overall, the spectra of PMs can be interpreted as a superposition of the raw materials, and no interaction could be detected between the compounds. To examine the changes occurring during the spray-drying process, the FT-IR spectra of the PMs and the SD samples were compared. To study interactions with IBU, it is crucial to first identify its most characteristic peak in the spectrum [38]. This peak appears as a sharp band at 1719 cm^−1^, corresponding to the C=O stretching vibrations of the carboxylic acid group. Subsequently, it was crucial to analyze the changes in this peak. When examining the PMs, the characteristic peak from the -COOH group of IBU was observed at the same positions, although in the case of SBECD, this was more difficult to identify, as it merged with the absorption originating from SBECD [31,73]. For the SD samples, this peak showed a significant reduction in intensity and a slight shift towards higher wavenumbers, indicating an interaction between IBU and the excipients [38]. For better clarity, we have highlighted these changes in Figure 9b. Based on the literature, IBU does not interact with MAN or LEU [17], suggesting that the observed interaction is specific to the CDs. This may be due to the weak interaction between the two components, just as the shift and reduction in the peak was possible due to the -COOH group being placed in the CD cavity [32,38,39,74]. Other studies have indicated that a shift in the IBU peak at 1508 cm^−1^ (ring vibration [31]) signifies interaction with the CD cavity [32]. However, no such shift was observed in our analysis; therefore, the observed changes are attributed to hydrophobic interactions between the CDs and IBU, rather than the formation of new intermolecular forces such as hydrogen bonding. We have recently published that the appearance of a new peak at around 1570 cm^−1^ suggests the presence of the ionized form of IBU, in addition to the absence of the signal corresponding to the -COOH group [18]. However, in the present study, no such signal indicating the ionized form of IBU was observed, implying that the active ingredient was present in the complex in its non-ionic form. The phenomenon was also indicated by the K value calculated from the phase solubility regarding the solution before the spray-drying process. This appearance may offer superior bioavailability compared to the ionic form. In summary, based on the changes observed in the FT-IR spectra, we concluded that the inclusion complex was formed, but there was no interaction between the active ingredient and the CDs.

### 2.5. In Vitro Dissolution and Diffusion Investigations

The bioavailability of IBU administered by the lungs is significantly influenced by the solubility and dissolution rate of the drug particles deposited in the pulmonary system. The in vitro dissolution and diffusion of reference IBU and the formulations exhibiting the highest FPF [%] values (SBECD-IBU-1MAN-0.5LEU SD and QABCD-IBU-1MAN-0.5LEU SD) were studied by simulating lung conditions.

**Figure 10:**
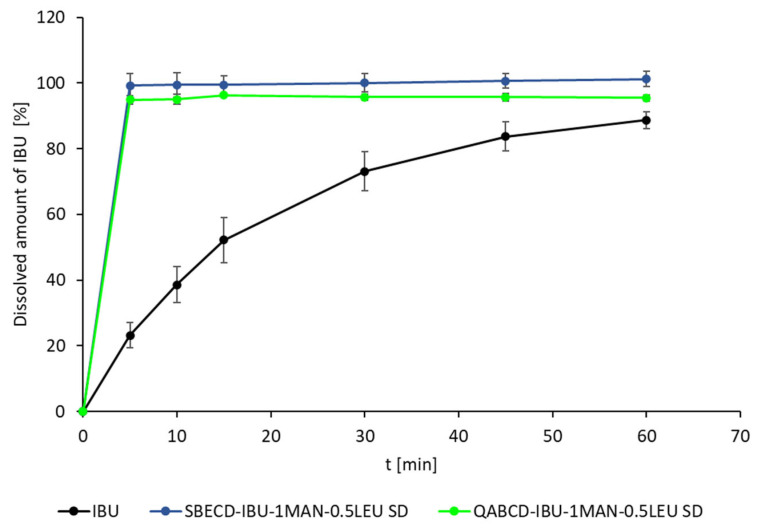
In vitro dissolution of IBU from SBECD and QABCD containing formulations compared to raw IBU in artificial lung fluid.

The results of in vitro dissolution tests were presented in Figure 10. The untreated IBU reached 88.7 ± 2.5% of its release over the investigated time period with slow dissolution. In contrast, both formulations containing CD dissolved the entire IBU within the first 5 min, while 101.2 ± 2.3% (SBECD-IBU-1MAN-0.5LEU SD) and 95.5 ± 1.0 (QABCD-IBU-1MAN-0.5LEU SD) of the drug was detected after this short time period. This indicates that by complex formulations, we significantly enhanced the solubility and dissolution rate of IBU. In comparison, only 23.1 ± 3.9% of the untreated IBU was dissolved in the first 5 min. The fast release profile of IBU from the complex could have been caused by several additional reasons in addition to complexation [75]. The amorphization of IBU also contributed to the improved dissolution behavior since amorphous compounds typically exhibit enhanced solubility and dissolution rates compared to their crystalline counterparts [76]. Moreover, the complexation of IBU with CDs, which alters its physicochemical properties, enhances its wettability [77,78]. This modification improves the drug’s interaction with the solvent, further promoting rapid dissolution. These variables play a crucial role in the fast dissolution observed in this study. In summary, the dissolution experiments demonstrated that complexation with CDs effectively enhanced the dissolution rate of IBU under pulmonary conditions.

**Figure 11:**
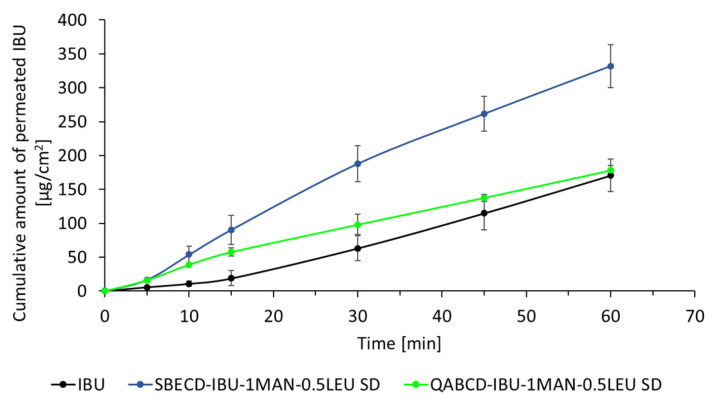
In vitro permeation of IBU from SBECD- and QABCD-containing formulations compared to raw IBU in artificial lung fluid.

A modified horizontal diffusion model was applied to study the in vitro permeability of IBU. The results of the in vitro permeation investigations are shown in Figure 11. The results indicate that the diffusion of IBU from the SBECD complex was enhanced compared to both untreated IBU and the QABCD complex; consequently, the dissolution rate did not affect the permeation rate as reported by Varga et al. in the case of the meloxicam potassium model drug [8]. Specifically, after 60 min, the cumulative permeated amount of IBU from the SBECD-IBU-1MAN-0.5LEU SD formulation reached 332 µg/cm^2^, whereas the corresponding values for untreated IBU and the QABCD-IBU-1MAN-0.5LEU SD formulation were 171 µg/cm^2^ and 178 µg/cm^2^, respectively. However, the initial dissolution rate was higher from the formulations with QABCD, despite the overall lower permeation. Inferring from the results of the cumulative amount of permeated drug, not surprisingly, the J and Kd values also showed a similar trend for the investigated materials. In the case of the SBECD-IBU-1MAN-0.5LEU SD, we achieved an outstandingly high diffusion rate, since J was 332 μg/(cm^2^⋅h) and Kp was 0.600 cm/h. These values were 178 μg/(cm^2^⋅h) and 0.374 cm/h for QABCD-IBU-1MAN-0.5LEU SD and 171 μg/(cm^2^⋅h) and 0.304 cm/h for IBU, respectively. The lack of IBU permeation from the QABCD-IBU-1MAN-0.5LEU SD complex compared to SBECD-IBU-1MAN-0.5LEU SD can be attributed to the formation of attractive electrostatic interactions between the cationic QABCD and the negatively charged IBU under physiological lung conditions. These interactions prevent the dissociation of the complex, thereby hindering its ability to pass through the apolar membrane as effectively as the IBU from the SBECD-containing complex. Despite the high FPF% and a dissolution rate comparable to the SBECD-IBU-1MAN-0.5LEU SD complex, the in vivo therapeutic efficacy of the QABCD-IBU-1MAN-0.5LEU SD formulation could be limited, as the IBU from this formulation may exhibit limited diffusion and membrane permeability, thereby reducing its absorption and subsequent therapeutic effect.

## 3. Materials and Methods

### 3.1. Materials

**Figure 12:**
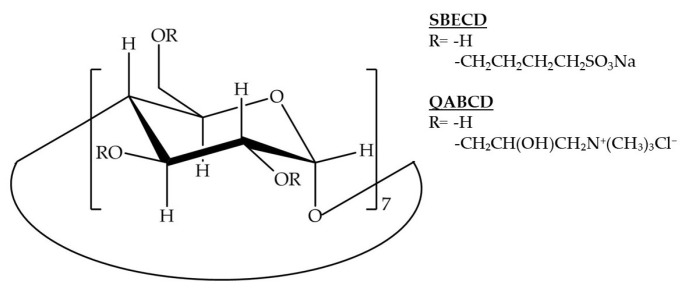
Structural formulas of SBECD and QABCD, the CD derivatives used in this study.

SBECD (DS~6.0, 5.8% water content) and QABCD (DS~3.3, 2.0% water content) were generously donated by Cyclolab R&D Laboratory Ltd. (Budapest, Hungary). The structural formulas of the CDs used are shown in Figure 12. Sanofi (Veresegyház, Hungary) provided the active ingredient IBU. To improve aerodynamic properties, excipients MAN (D-mannitol, Molar Chemicals Kft, Halásztelek, Hungary) and LEU (L-leucine, AppliChem GmbH, Darmstadt, Germany) were used. Purified water was used as a solvent. Simulated lung fluid contained 0.68 g/L NaCl, 2.27 g/L NaHCO_3_, 0.02 g/L CaCl_2_, 0.1391 g/L NaH_2_PO_4_, 0.37 g/L glycine, and 5.56 mL/L of 0.1 M H_2_SO_4_ in water solvent. All reagents and solvents were used without further purification. Phosphate buffer solution (PBS, pH = 3.0) and acetonitrile of HPLC grade (LGC Promochem, Wesel, Germany) were used for high-pressure chromatography.

### 3.2. Methods

#### 3.2.1. Preparation of the Samples

##### Phase Solubility Study

In order to select the concentration of the CD solutions used and the equilibrium molar ratio of CD/IBU, a phase solubility study was performed. The phase solubility study was conducted in a manner analogous to the method described in the literature [1,79]. Accordingly, an excess amount of IBU was added to CD solutions (SBECD and QABCD) of different concentrations (between 5 and 25 mM). The prepared samples were mixed at 100 rpm for 24 h at 25 °C. After equilibrium followed by centrifugation and filtration with a 0.45 mm PVDF syringe filter, the concentration of the prepared solutions was determined by HPLC. Using Equations (1)–(3), the stability constant (K), the complexation efficiency (CE), and the drug/CD molar ratio of the complexes were calculated, respectively.

(1) $$K=\frac{\mathrm{Slope}}{S_{0}\left(1-\mathrm{Slope}\right)}$$

(2) $$\mathrm{CE}=\frac{\mathrm{Slope}}{1-\mathrm{Slope}}$$

(3) $$\mathrm{drug}\text{:}\mathrm{CD}\;\mathrm{molar}\;\mathrm{ratio}=1\text{:}\frac{\mathrm{CE}+1}{\mathrm{CE}}$$

where S_0_ is the intrinsic solubility of IBU in the absence of SBECD or QABCD in water, although the slope is obtained from the plot of IBU versus SBECD or QABCD molar concentration.

##### Preparation of the Nano-Spray-Dried Particles

**Figure 13:**
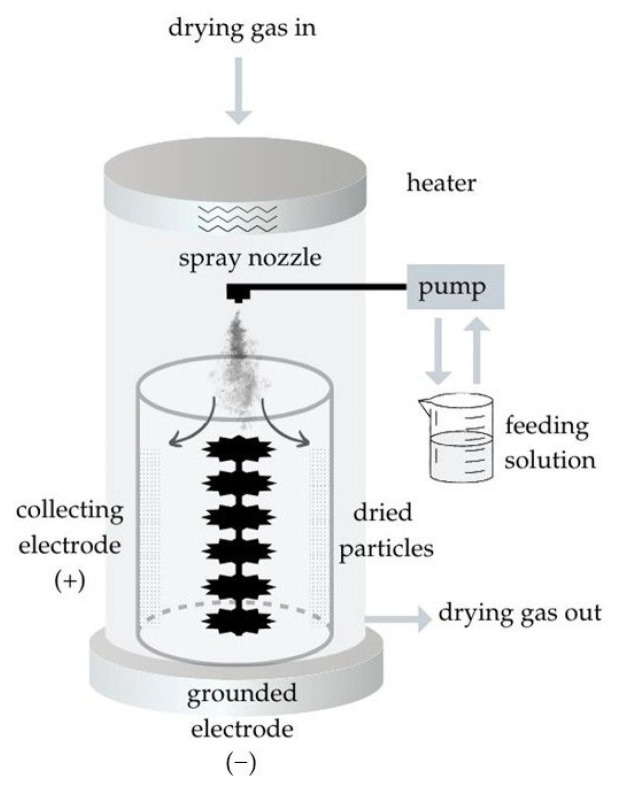
Schematic representation of the nano-spray-dryer.

DPIs were prepared using a BÜCHI Nano Spray Dryer B-90 HP (BÜCHI Labortechnik AG, Flawil, Switzerland). The instrument, based on a novel spray-drying concept, utilizes advanced vibrating mesh technology to generate significantly smaller droplets than conventional spray-dryers. A schematic representation of the nano-spray-dryer is shown in Figure 13. This innovation enables the production of submicron-sized particles with narrow size distributions and high yields [80]. The process parameters were set as follows: 70 °C inlet air temperature, 20% pump speed, 100% aspirator capacity, and 130 L·h^−1^ compressed air flow rate. The phase solubility results were used to determine the concentration of SBECD and QABCD and the molar ratio of IBU to CDs. The ratio of MAN and LEU was compared to the weight of IBU, and their values were selected based on the work published by M. Malamatari et al. [54]. The composition of the nano-spray-dried samples is presented in Table 5 in the case of SBECD. Among the listed combinations, the sample with outstanding aerosolization properties was also prepared and examined with QABCD later.

The physical mixtures (PMs) were prepared for the investigations as references in the same ratio as the solutions of the nano-spray-dried samples. The compounds were mixed in a Turbula mixer (Turbula WAB, Systems Schatz, Muttenz, Switzerland) for 10 min at 50 rpm.

#### 3.2.2. Investigation of the Nano-Spray-Dried Particles

##### Investigation of the Particle Size of the Nano-Spray-Dried Formulations by Laser Diffraction

The particle size and particle size distribution of the samples were determined using a laser diffraction method with the Malvern Mastersizer Scirocco 2000 (Malvern Instruments Ltd., Worcestershire, UK). Dry dispersion equipment was utilized with air as the dispersion medium, operating at a pressure of 3.0 bar and 75% vibration feed. Each type of sample was analyzed in triplicate. The particle size distribution was characterized using the D [0.1], D [0.5], and D [0.9] values, representing the diameters below 10%, 50%, and 90% of the particle volume distributed, respectively. Additionally, the span value was calculated to assess the width of the distribution based on Equation (4):

(4) $$\mathrm{Span}=\frac{D\left[0.9\right]-D\;\left[0.1\right]}{D\;\left[0.5\right]}$$

Refractive indices were set according to the sample type, with values of 1.44 and 1.45 for SBECD- and QABCD-based samples, respectively.

By using laser diffraction measurements, we were able to choose formulations that satisfied the requirements for pulmonary delivery, such as having an average diameter of less than 10 µm and a narrow particle size range.

##### In Vitro Aerodynamic Investigation

**Figure 14:**
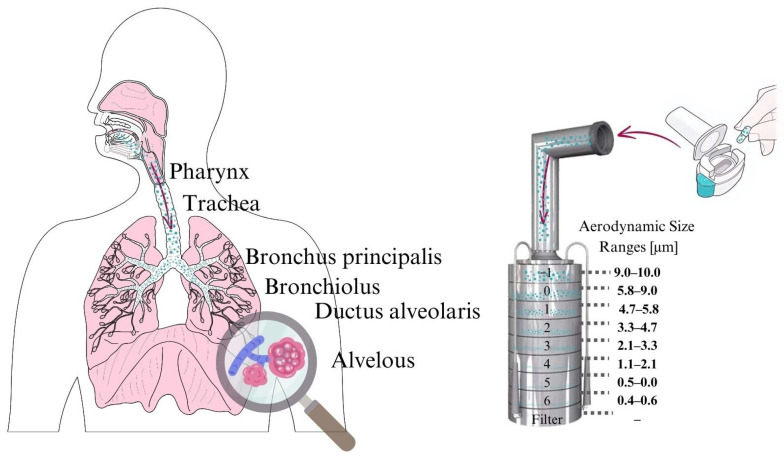
Schematic representation of the working principle of the Andersen Cascade Impactor.

The in vitro aerodynamic properties of the nano-spray-dried formulations were evaluated using an Andersen Cascade Impactor (Figure 14) Apparatus D, Copley Scientific Ltd., Nottingham, UK. The inhalation flow rate was set to 60 L/min and confirmed with a Critical Flow Controller Model TPK and a High-capacity Pump Model HCP5. Each inhalation lasted 4 s, simulating a 4 L breathing volume. Breezhaler^®^ single-dose devices and hydroxypropyl methylcellulose capsules (size 3 Ezeeflo^TM^, ACG-Associated Capsules Pvt. Ltd., Mumbai, India) were used for inhalation, filled with a constant powder mass. The amount of IBU in the in vitro tests was 5 mg, as previously reported [18]. Each sample was tested in triplicate. The stages of the impactor were coated with a cyclohexane and Span 85 (1 *w*/*w*%) mixture to retain particles. After inhalation, the device, capsule, induction port, collection plates, and filter were washed with water to collect the deposited IBU. The active ingredient was quantified by HPLC measurements. FPF and MMAD values were calculated by the Inhalytix^TM^ software v 2.0 from Copley Scientific Ltd., Nottingham, UK, to characterize aerodynamic performance. FPF represented the percentage of particles with an aerodynamic diameter below 5 µm relative to the emitted dose. MMAD was determined from the aerodynamic particle size distribution.

##### Determination of Concentration with High-Performance Liquid Chromatography (HPLC)

The concentration of IBU was determined using an Agilent 1260 HPLC system (Agilent Technologies, Santa Clara, CA, USA) equipped with a UV–VIS diode array detector, based on our previously published method for IBU determination [18]. A reversed-phase Kinetex^®^ EVO C18 column (5 mm, 150 mm × 4.6 mm (Phenomenex, Torrance, CA, USA)) was the stationary phase, and the column temperature was set to 25 °C. The mobile phase contained PBS with pH = 3.0 (A) and acetonitrile (B). Isocratic elution was applied with 45–55% A–B eluent composition for 6 min at a flow rate of 1 mL·min^−1^. IBU concentration was measured at 220 nm using a UV–VIS diode array detector. The retention time of IBU was 4.09 min.

##### In Silico Characterization

The in silico simulations were conducted using a stochastic lung deposition model, which tracks inhaled particles until their deposition or exhalation and calculates the fraction of particles deposited in different anatomical regions of the respiratory system [64,81]. Particle trajectories were simulated within an asymmetrical branching airway structure, designed to replicate realistic airway morphology through the selection of appropriate morphometric parameters. In this study, the aerodynamic particle size distribution of the samples, measured using the Andersen Cascade Impactor, was used as input for the airway deposition model. Simulations were performed with two different breath-holding times (5 and 10 s) to evaluate their effects on particle deposition. The computational model had been previously validated in earlier studies involving aerosol drug delivery, ensuring its reliability for this investigation [82,83].

##### Morphological Examination of the Samples with Scanning Electron Microscopy (SEM)

The samples surface characteristics were analyzed using SEM (Hitachi S4700, Hitachi Scientific Ltd., Tokyo, Japan). During the measurements, the following parameters were used: 10 mA amperage, 10 kV high voltage, and 1.3–13.1 mPa air pressure under an argon atmosphere. The powders were coated with gold–palladium to make them conductive.

##### Examination of the Crystallinity of the Formulations by X-Ray Powder Diffraction (XRPD)

The prepared sample’s structure was assessed using a BRUKER D8 Advance X-Ray Powder Diffractometer (Bruker AXS GmbH, Karlsruhe, Germany). The tube anode was Cu with Kα radiation (λ = 1.5406 Å), and the line detector a VÅNTEC-1. The measurement was performed with the following settings: at 40 kV and 40 mA within an angular range of 3° to 40° 2θ, with a step time of 0.1s and a step size of 0.01°. We manipulated the acquired data (Kα2-stripping, background removal, and smoothing) using DIFFRACplus EVA software (version 5.2).

##### Examination of Thermal Properties with Differential Scanning Calorimetry (DSC)

In order to examine the thermal changes of IBU in the formulations, DSC measurements were conducted. The thermal analysis was performed using the Mettler Toledo DSC/TGA 821e system (Mettler Toledo Inc., Schwerzenbach, Switzerland) and evaluated with the STARe software (Version 9.1, Mettler Toledo Inc., Schwerzenbach, Switzerland). Approximately 2–5 mg of samples was placed in sealed and perforated aluminum pans and heated from 25 °C to 300 °C at a heating rate of 10 °C·min^−1^. The analysis was carried out under a constant argon flow of 10 L·h^−1^.

##### Investigation of Chemical Interactions with Fourier-Transformed Infrared Spectroscopy (FT-IR)

The interactions between nano-spray-dried CD/IBU complexes were investigated using an AVATAR330 FT-IR spectrometer (Thermo Nicolet, Unicam Hungary Ltd., Budapest, Hungary) in the interval of 400–4000 cm^−1^, at an optical resolution of 4 cm^−1^. Samples were ground and compressed into pastilles at 10 t with 0.15 g of KBr. Spectragryph optical spectroscopy software v.1.2.16.1 was used to provide baseline correction and peak normalization for the purpose of evaluating spectra.

##### In Vitro Dissolution Measurement

We applied the dissolution test using the previously published technique [18] by the research group since there are no formal regulatory standards or guidelines for the in vitro dissolution testing of inhalation products [44]. According to this, the dissolution profile of IBU was examined in vitro using a modified approach. According to this, the dissolution medium was 20 mL of 37 °C, 7.4 ± 0.1 pH artificial lung fluid, which was stirred with a magnetic stirrer at 100 rpm. The same amounts as the doses investigated with the Andersen Cascade Impactor were measured for the dissolution test. Samples of 1 mL were taken at 5, 10, 15, 30, 45, and 60 min intervals. The release was repeated three times for each sample. The samples were filtered with a 0.22 µm pore size syringe filter, then the quantitative determination was performed by HPLC measurements.

##### In Vitro Permeability Measurement

**Figure 15:**
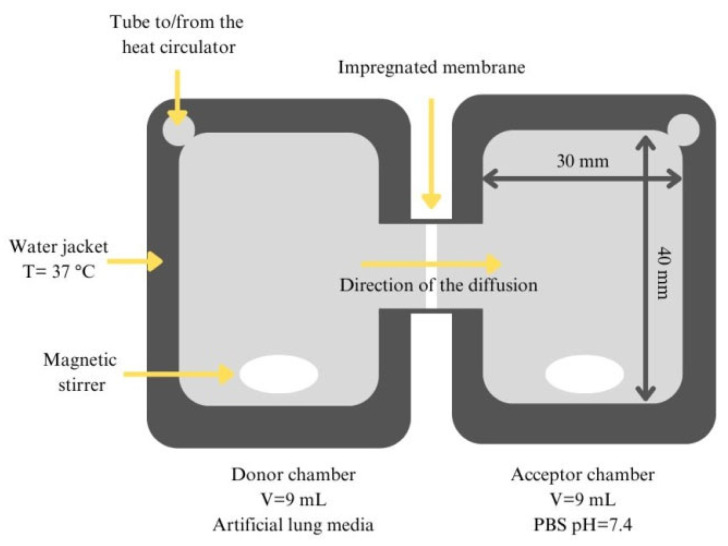
The schematic setup of the modified horizontal device used for the in vitro diffusion test.

A modified horizontal diffusion model (Figure 15) was applied to study the in vitro permeability of IBU [84]. The device consisted of two chambers, one serving as the donor phase and the other as the acceptor phase, separated by an artificial membrane. The donor phase contained a simulated lung medium, while the acceptor phase used a phosphate buffer (pH 7.4) to imitate physiological conditions. The membrane used in this study was a regenerated cellulose filter (Whatman^TM^) with a pore size of 0.45 µm, pre-soaked in isopropyl myristate to simulate lipophilic biological membranes. The system was maintained at a controlled temperature (37 °C) and stirred continuously at 300 rpm to ensure homogeneity. Samples containing 5 mg of IBU were introduced into the donor phase, and the cumulative permeation was measured over time using the HPLC method. The permeability parameters were calculated using equations involving the flux (J) and the permeability coefficient (Kp). These parameters are described by Equation (5) and Equation (6), respectively.

(5) $$J=\frac{m}{A\times t}$$

(6) $$\mathrm{Kp}=\frac{J}{C}$$

##### Statistical Analysis

To evaluate whether there were statistically significant differences among the measured values, statistical analysis was conducted. For the in vitro cascade impactor and in silico tests, a one-way ANOVA followed by a Tukey HSD post hoc test was used. *p*-values < 0.05 indicated statistically significant differences.

## 4. Conclusions

In this study, CD/IBU complexes were prepared using a nano-spray-drying technique. Based on the phase solubility study, we determined the CD concentration together with the CD/IBU molar ratio for complete complex formation. The CD concentration was set at 15 mM, while the CD/IBU ratio was 1.3:1.0 mol. The aerosolization properties of SBECD- and QABCD-based formulations were successfully enhanced by optimizing the ratio of MAN and LEU excipients. The most outstanding results were achieved with formulations in which the weight ratios of MAN and LEU to IBU were 1.0 and 0.5. Namely, the FPF value of the SBECD-IBU-1MAN-0.5LEU SD formulation became 50.05 ± 1.05%, while in the case of QABCD-IBU-1MAN-0.5LEU SD, this value was 51.01 ± 4.06%. Based on morphological studies, we concluded that the small amount of MAN and LEU compared to the total weight of the formulations can significantly influence the surface structure of the particles. According to the characterization of samples, we found that the spray-dried samples were amorphous, which is particularly beneficial for pulmonary administrations, as the resulting increased dissolution rate significantly enhances bioavailability, thereby improving the therapeutic efficacy of the formulation. The amorphization was also confirmed by DSC measurements and indicated the thermal stability of the complexes. No evidence of intermolecular interactions was observed. The in vitro dissolution and diffusion studies revealed rapid IBU release from the formulations; however, the QABCD-based sample exhibited reduced membrane diffusion compared to SBECD due to the development of electrostatic interactions. In summary, the resulting formulations demonstrated suitability for pulmonary drug delivery as DPIs, with potential application in cystic fibrosis treatment. Since inflammation is a major contributor to pulmonary complications in cystic fibrosis, anti-inflammatory therapy can help slow disease progression. Clinical studies have confirmed the efficacy of long-term, high-dose IBU therapy in cystic fibrosis patients. Therefore, our preformulation may offer a promising therapeutic approach in the management of cystic-fibrosis-related lung inflammation.

## Figures and Tables

**Table 1:** K, CE, and CD/IBU molar ratio calculated from phase solubility results.

	SBECD	QABCD
K [M^−1^]	9753	9521
CE	3.58	3.49
IBU:CD	1:1.27	1:1.29

**Table 2:** d(0.1), d(0.5), d(0.9), and span values based on laser diffraction measurements. Data are presented as means ± SD (*n* = 3 independent measurements).

	D(0.1) [μm]	D(0.5) [μm]	D(0.9) [μm]	Span
SBECD-IBU SD	1.0 ± 0.1	2.5 ± 0.1	28.6 ± 11.6	11.6 ± 11.1
SBECD-IBU-1MAN-0.5LEU SD	1.2 ± 0.2	3.1 ± 1.1	32.5 ± 2.1	10.8 ± 4.6
SBECD-IBU-3MAN-0.5LEU SD	1.7 ± 0.1	4.2 ± 2.6	148 ± 164.6	164.6 ± 16.9
SBECD-IBU-1MAN-1.5LEU SD	1.3 ± 0.1	2.9 ± 0.6	23.8 ± 22.9	2.9 ± 1.4
SBECD-IBU-3MAN-1.5LEU SD	1.3 ± 0.2	3.3 ± 0.7	54.4 ± 22.9	17.2 ± 11.3

**Table 3:** Summary of the aerodynamic characteristics of the nano-spray-dried powders with SBECD. Data are means ± SD (*n* = 3 independent measurements).

Sample	FPF [%]	MMAD [µm]
SBECD-IBU SD	15.11 ± 7.54	no data
SBECD-IBU-1MAN-0.5LEU SD	50.05 ± 1.05	3.83 ± 0.19
SBECD-IBU-3MAN-0.5LEU SD	19.46 ± 1.02	7.98 ± 0.68
SBECD-IBU-1MAN-1.5LEU SD	35.70 ± 1.39	5.14 ± 0.26
SBECD-IBU-3MAN-1.5LEU SD	40.07 ± 2.02	4.65 ± 0.18

**Table 4:** The aerodynamic parameters of samples with and without aerosolization excipients in the case of different CDs. Data are means ± SD (*n* = 3 independent measurements).

Sample	FPF [%]	MMAD [µm]
SBECD-IBU SD	15.11 ± 7.54	no data
QABCD-IBU SD	35.20 ± 9.42	5.19 ± 1.98
SBECD-IBU-1MAN-0.5LEU SD	50.05 ± 1.05	3.83 ± 0.19
QABCD-IBU-1MAN-0.5LEU SD	51.01 ± 4.06	4.10 ± 0.16

**Table 5:** Composition of the nano-spray-dried samples.

Sample	Distilled Water [mL]	SBECD [mg]	IBU [mg]	MAN [mg]	LEU [mg]
SBECD-IBU	10	331.88	23.80	–	–
SBECD-IBU-1MAN-0.5LEU-SD	10	331.88	23.80	23.80	11.90
SBECD-IBU-3MAN-0.5LEU SD	10	331.88	23.80	71.41	11.90
SBECD-IBU-1MAN-1.5LEU SD	10	331.88	23.80	23.80	35.70
SBECD-IBU-3MAN-1.5LEU SD	10	331.88	23.80	71.42	35.70

## Data Availability

Data are contained within the article.
